# Super Broadband at Telecom Wavelengths From RE^3+^-Doped SiO_2_-Ta_2_O_5_ Glass Ceramics Planar Waveguides

**DOI:** 10.3389/fchem.2022.915335

**Published:** 2022-07-04

**Authors:** Karmel De Oliveira Lima, Fábio José Caixeta, Vítor Dos Santos De Souza, Leonardo Sousa Rosa, Victor Del Vecchio Soares, Carolina Assunção Crumo, Ivana Aparecida Borin, Jefferson Luis Ferrari, Rogéria Rocha Gonçalves

**Affiliations:** ^1^ Laboratório de Materiais Luminescentes Micro e Nanoestruturados—Mater Lumen, Departamento de Química, Faculdade de Filosofia Ciências e Letras de Ribeirão Preto, Universidade de São Paulo, Ribeirão Preto, Brazil; ^2^ Laboratório de Desenvolvimento de Materiais Inorgânicos com Terras Raras–DeMITeR, Instituto de Química, Universidade Federal de Uberlândia, Uberlândia, Brazil

**Keywords:** NIR emission, broad band, waveguide, glass ceramic, rare earth

## Abstract

This paper reports on the preparation of Er^3+^/Yb^3+^/Tm^3+^, Er^3+^/Yb^3+^/Nd^3+^, and Er^3+^/Tm^3+^/Nd^3+^ triply doped and Er^3+^-doped SiO_2_-Ta_2_O_5_ glass ceramic nanocomposites and active planar waveguides by the sol–gel process using the dip-coating technique as deposition method. The investigation of their structural, morphological, and luminescent properties using XRD, AFM, and photoluminescence analysis, are reported here. The XRD results showed the presence of L-Ta_2_O_5_ nanocrystals dispersed in the SiO_2_-based amorphous host for all the nanocomposites and films. The rare earth ion (RE^3+^) doping concentration affected both the crystallinity, and the crystallite sizes of the Ta_2_O_5_ dispersed into SiO_2_-Ta_2_O_5_ nanocomposites and waveguides. AFM characterization revealed crack free and smooth surface roughness and differences in viscoelasticity on the Er^3+^-doped SiO_2_-Ta_2_O_5_ films surface, which allows the identification of Ta_2_O_5_ nanocrystals on the SiO_2_ amorphous host. The Er^3+^ doped and triply doped SiO_2_-Ta_2_O_5_ nanocomposites displayed broad- and super broadband NIR emissions with a FWHM up to 173 nm achieved in the telecom wavelengths. The lifetime of the ^4^I_13/2_ emitting level of the Er^3+^-doped SiO_2_-Ta_2_O_5_ waveguides is strongly dependent on Er^3+^ concentration and an emission quenching was negligible up to 0.81 mol%. The structural and luminescent investigations indicated that RE^3+^-doped SiO_2_-Ta_2_O_5_ glass ceramics are promising candidates for photonic applications in optical devices operating in wide wavelengths at the telecom bands.

## Introduction

The global internet traffic has greatly grown over the last years, for instance, by 2023 the forecasts have pointed out 5.3 billion users, 29.3 billion networked devices, 14.7 billion machine-to-machine connections, and global fixed broadband speeds will reach 110.4 Mbps (Cisco, 2020). Moreover, mainly due to the Coronavirus disease (COVID-19) pandemic it going to keep increasing. Home office, online learning, streaming services, e-commerce, delivery, social media platforms, gaming, and so on have boosted the global internet consuming. Indeed, according to Packet Clearing House (PCH) the domestic bandwidth production has soared worldwide over the last year (PCH, 2021). In this context, one of the most pressing technological challenges to overcome nowadays is the ever-increasing demand for telecommunication networks that can carry larger data volumes. Therefore, developing efficient new photonic materials for telecommunications is imperative.

Since the 1980s the optical fibers and erbium doped fiber amplifiers (EDFAs) that work at 1.5 μm region have played an important role concerning high transmission rate, large capacity and low-losses of signal transmission in the so called third telecommunication window (Mears et al., 1987; Desurvire et al., 1987). Notwithstanding, erbium doped planar waveguide amplifiers (EPWAs) have been successfully used as efficiency platforms for optical information transport in integrated optics (IO) (Ferrari et al., 2021).

Erbium (Er^3+^) ion doped materials are very used as optical amplifiers for operation in the S, C, and L telecom bands (1460–1610 nm), depending fundamentally on the chemical composition of the matrix.

An important aspect to be consider in telecommunication is bandwidth. Over the past decades, much attention has been dedicated to obtaining luminescent materials that emit broad emission bands in the near infrared (NIR) region for optical amplification. Several strategies concerning hosts and doping parameters have been adopted to enlarge NIR emission not only in the S, C, and L, but also O, E, and U telecom bands. Among the many strategies developed for the preparation of superbroadband optical amplifiers reported in the literature, special attention has been devoted to glassy and glass-ceramic materials doped with bismuth ion (Bi^+^) and several rare earth ions (RE^3+^) (Rivera et al., 2014; Sun et al., 2014; Martins et al., 2019; Thipparapu et al., 2019).

The use of several RE^3+^ dopants in waveguide materials proposes the widest gain amplification spectrum, increasing the transmission capacity of them. Thulium (Tm^3+^) doped amplifiers can operate in the S-band (1460–1530 nm), as well in the region from 1.2 to 2 μm, increasing the transmission via shifting away from the third telecommunication window (Muscelli et al., 2016; Aquino et al., 2018; Khamis and Ennser, 2019). Neodymium (Nd^3+^) single doped materials show one near-infrared band emission at 1.3 µm, which is placed in the O- and E-bands (Herrera et al., 2021; Xia et al., 2021). The combination of different RE ions in the same host, allows the wide or ultra-wide band optical amplifier development which could span most part of the low-loss region of the main telecommunication window (Shen et al., 2020b).

A key point concerning the development of photonic materials doped with rare earth (RE) ions is the choice of the host lattice. In this sense, refractive index, phonon energy, RE solubility, symmetry sites, emission quenchers, optical transparency, and chemical stability are the most critical factors for the development of photonic devices with broadband emissions.

Several matrices have been investigated for photonic applications, especially for optical amplification, such as RE^3+^ doped tellurite glasses due to them lower phonon energies (Huang et al., 2004; Shen et al., 2020b), phosphate glasses due to them mechanical strength, good thermal stability and excellent flexibility (Li et al., 2016). The RE^3+^ ions combinations with other ones (Bi^3+^, Bi^+^, Cr^3+^, …) in glasses and glass-ceramics, have been studied in the last decades (Meng et al., 2005; Yang et al., 2018; Dan et al., 2020).

Among the potential RE doped matrices for telecommunication, tantalum oxide (Ta_2_O_5_) has attractive properties like low phonon energy (<700 cm^−1^), transparency over a wide spectral range from visible to NIR regions, high refractive index (n ∼ 2.15), chemical stability (Muscelli et al., 2016). Previous studies published by some of the authors have revealed that Er^3+^ doped SiO_2_-Ta_2_O_5_ glass ceramics can be potentially used in EDWA and WDM devices (Ferrari et al., 2010), showing NIR emission with bandwidth values from 65 to 91 nm, indicating that Er^3+^ ions are preferentially placed in the Ta_2_O_5_ sites due to the complex structure of Ta_2_O_5_ (Ferrari et al., 2011a).

Materials with potential applications as active planar waveguides have played an important role in the development of optics and optoelectronics applications due to their excellent performance, low cost, and ease of fabrication.

Planar waveguides have to combine several requirements in order to achieve high light confinement coefficient and low propagation losses, such as high control of refractive index values, absence or high control of phase separation, high solubility of lanthanide ions (Terui and Kobayashi, 1978; Benatsou et al., 1997) among others.

One of the best and low cost experimental routes to the manufacture of multicomponent glass ceramics and planar waveguides is the sol-gel process (Benatsou et al., 1997; Gonçalves et al., 2002, 2004). This methodology presents several advantages in comparison with melt-quenching technique, being considered an adequate method to control all the requirements mentioned above, considering that such a process allows the possibility of synthesis of multicomponent systems, high homogeneity, among other factors.

This study reports on the synthesis, structural and morphological characterization, and luminescent properties of rare earth ion (RE^3+^)-doped SiO_2_-Ta_2_O_5_ glass ceramic nanocomposites and active planar waveguides prepared by the sol–gel methodology. More specifically, we have prepared photonic glass ceramic materials with several Er^3+^ concentrations and different RE^3+^ doping combinations to widen the near infrared (NIR) emission band and consequently enable the application in a wider region in the window used in telecommunications. The preparation of materials with broad- and super broadband opens the possibility of manufacturing integrated optics components such as optical amplifiers based on RE^3+^ -doped planar waveguide amplifiers operating in a wide range of wavelengths for new concept of optical devices.

## Materials and Methods

### Materials

Er_2_O_3_ (99.9%), Yb_2_O_3_ (99.9%), Tm_2_O_3_ (99.9%), Nd_2_O_3_ (99.9%), tantalum ethoxide (99.98%), 2-ethoxyethanol (99%) and tetraethylorthosilicate (TEOS, 98%) were purchased from Sigma Aldrich. Anhydrous ethanol (≤0.005% H_2_O) was obtained from Merck. HCL PA-ACS-ISO (37wt%) were acquired from Panreac.

### Synthesis of Materials

Er^3+^/Yb^3+^/Tm^3+^, Er^3+^/Yb^3+^/Nd^3+^, Er^3+^/Tm^3+^/Nd^3+^ triply doped and Er^3+^-doped SiO_2_-Ta_2_O_5_ nanocomposites and films were prepared by the sol–gel methodology. First, the Er^3+^, Yb^3+^, Tm^3+^, and Nd^3+^ chloride ethanolic solutions were prepared by dissolving Er_2_O_3_, Yb_2_O_3_, Tm_2_O_3_, and Nd_2_O_3_, respectively, in HCl PA-ACS-ISO under stirring. The solutions were subsequently dried at 80°C with addition of anhydrous ethanol. Finally, the resulting dopant solutions were titrated by ethylenediaminetetraacetic acid (EDTA, 0.0515 mol L^−1^) complexation in Acetic Acid-Sodium Acetate buffer (pH 5.4); xylenol orange was used as indicator.

A final reaction volume of 20.0 ml, with total Si + Ta concentration of 0.448 mol L^−1^ and Si/Ta 70:30 molar ratio, was prepared. In a container under stirring at room temperature, anhydrous ethanol, tantalum ethoxide, 2-ethoxyethanol, and the dopant solutions were added. In a seconder container under stirring at room temperature, anhydrous ethanol, tetraethylorthosilicate, and HCl PA-ACS-ISO were added at TEOS/HCl 50:1 ratio. Then, the contents of the two containers were mixed under stirring at room temperature for 30 min. The obtained sols were used to prepare the films, as described in detail elsewhere (Ferrari et al., 2010), or dried for 60 days for xerogel formation. The resulting xerogels were ground to powder and annealed at 900 and 1100°C for 8 h. Table 1 summarizes the prepared samples.

**TABLE 1:** Triply doped and Er^3+^-doped SiO_2_-Ta_2_O_5_ nanocomposites and films.

Dopant (mol%)	Sample processing
0.5Er^3+^/3.0Yb^3+^/1.0Tm^3+^	Powder
0.5Er^3+^/3.0Yb^3+^/1.0Nd^3+^	Powder
0.3Er^3+^/0.9Tm^3+^/0.05Nd^3+^	Powder
0.03Er^3+^	Powder and film
0.1Er^3+^	Powder and film
0.3Er^3+^	Powder and film
0.5Er^3+^	Powder and film
1.0Er^3+^	Powder and film
2.0Er^3+^	Powder and film
4.0Er^3+^	Powder and film

### Characterization

The powder X-ray diffraction (XRD) measurements of the triply doped SiO_2_-Ta_2_O_5_ nanocomposites were carried out on a Siemens-Bruker D5005-AXS diffractometer operating with CuKα (40 kV and 30 mA) radiation (1.5406 Å) and equipped with a graphite monochromator. The powder XRD patterns were recorded at 0.02°s^−1^, in the 2θ range between 5 and 90°. As for the powder XRD patterns of the Er^3+^-doped SiO_2_-Ta_2_O_5_ powders and films, they were registered in the 2θ range between 15 and 70°. The crystallite sizes were determined by the Scherrer formula (Patterson, 1939). The calculation was based on the measurement of integral breadth values in the corresponding XRD pattern. The planar waveguide surfaces were investigated by Atomic Force Microscopy (AFM) with a Shimadzu scanning probe microscope (SPM-9600) operating at the contact and dynamic modes. Surface roughness was evaluated by using the images collected with the software SPM, version 3.03.

The Photoluminescence (PL) emission spectra of the triply doped SiO_2_-Ta_2_O_5_ nanocomposites in the near infrared (NIR) region were recorded at room temperature on a FluoroLog-3 Horiba Jobin Yvon spectrofluorometer equipped with a H10330-75 NIR photomultiplier. The excitation sources were 808-nm and 980-nm diode lasers (DMC Group) operating at 1000 mW.

The PL emission spectra in the region between 1280–1700 and 1400–1680 nm of the Er^3+^-doped SiO_2_-Ta_2_O_5_ nanocomposites and the PL decay curves of the Er^3+^-doped SiO_2_-Ta_2_O_5_ waveguides were registered at room temperature on a Fluorolog 3-222 Horiba Jobin Yvon spectrofluorometer equipped with an InGaAs detector. The excitation source was a 980-nm laser operating at 302 mW (CrystaLaser). The emission decays of the ^4^I_13/2_ emitting level of Er^3+^ in the SiO_2_-Ta_2_O_5_ films were measured under excitation at the Er^3+^:^4^I_15/2_→^4^I_11/2_ transition at room temperature and recorded with an oscilloscope (Tektronix TDS 2022B).

## Results and Discussion

### Structural and Morphological Properties

**FIGURE 1:**
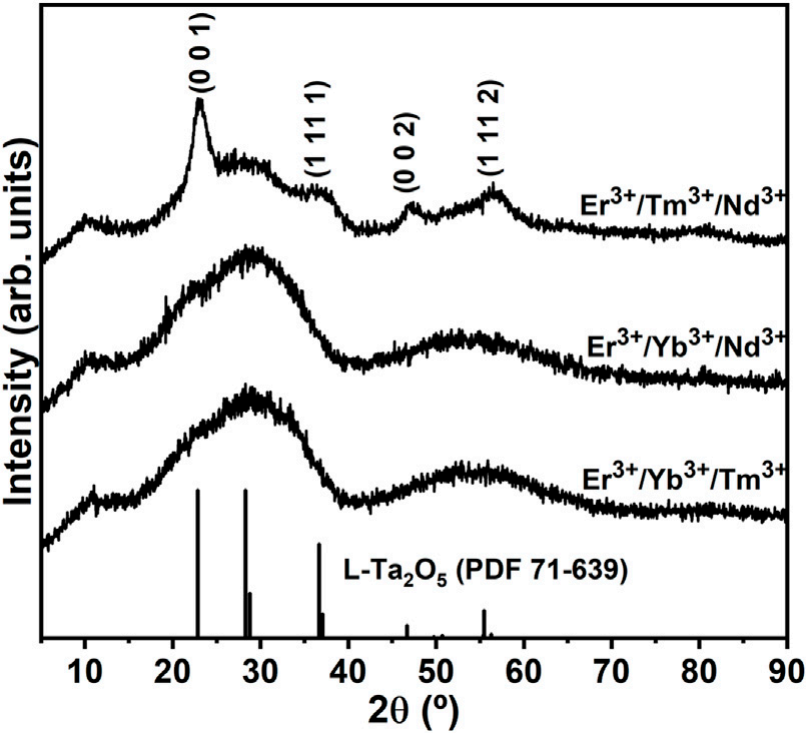
Powder XRD patterns of triply doped SiO_2_-Ta_2_O_5_ nanocomposites annealed at 900°C for 8 h.

Figure 1 presents the powder XRD patterns of the triply doped SiO_2_-Ta_2_O_5_ nanocomposites annealed at 900°C for 8 h. The powder XRD patterns of the Er^3+^/Yb^3+^/Tm^3+^ and Er^3+^/Yb^3+^/Nd^3+^ triply doped nanocomposites displayed an amorphous halo from 15° to 40°, and no crystallization peaks were detected. On the other hand, the Er^3+^/Tm^3+^/Nd^3+^ triply doped nanocomposite presented crystallization peaks at 22.8°, 36.7°, 46.7°, and 55.5°, assigned to the (0 0 1), (1 11 1), (0 0 2), and (1 11 2), respectively, crystalline planes of orthorhombic L- Ta_2_O_5_ (Stephenson and Roth, 1971). A full description and discussion of such crystalline phase can be seen elsewhere (Stephenson and Roth, 1971; Ferrari et al., 2011b). Herein, it is evidenced that Ta_2_O_5_ crystallization was significantly affected where the process was delayed for samples with the highest concentrations of RE^3+^ concentration (the total RE^3+^ concentration was 4.5, 4.5, and 1.25 mol% for the Er^3+^/Yb^3+^/Tm^3+^, Er^3+^/Yb^3+^/Nd^3+^, and Er^3+^/Tm^3+^/Nd^3+^ triply doped nanocomposites, respectively) in which the ions may be acting on the surface of the particles reducing the energy surface avoiding their increasing. This observation corroborates the indication that RE^3+^ ions will preferentially be distributed in an environment rich in tantalum oxide. With the formation of nanoparticles with controlled segregation, the distribution of lanthanide ions will be into the tantalum oxide nanocrystals, as observed in previous works. The RE^3+^ distribution in nanocrystals containing the complex structure of tantalum oxide has revealed the distribution of RE^3+^ ions in a wide range of slightly different symmetry sites, resulting in an inhomogeneous broadening of the emission band in the near infrared (Ferrari et al., 2011b).

One of our goals was to obtain a broad emission band around the 1.5-µm region, so inhomogeneous broadening was desirable due to the large number of symmetry sites occupied by RE^3+^ ions. In this sense, the reduction in crystallization can directly influence the inhomogeneous broadening of the NIR emission band, as we will see below.

**FIGURE 2:**
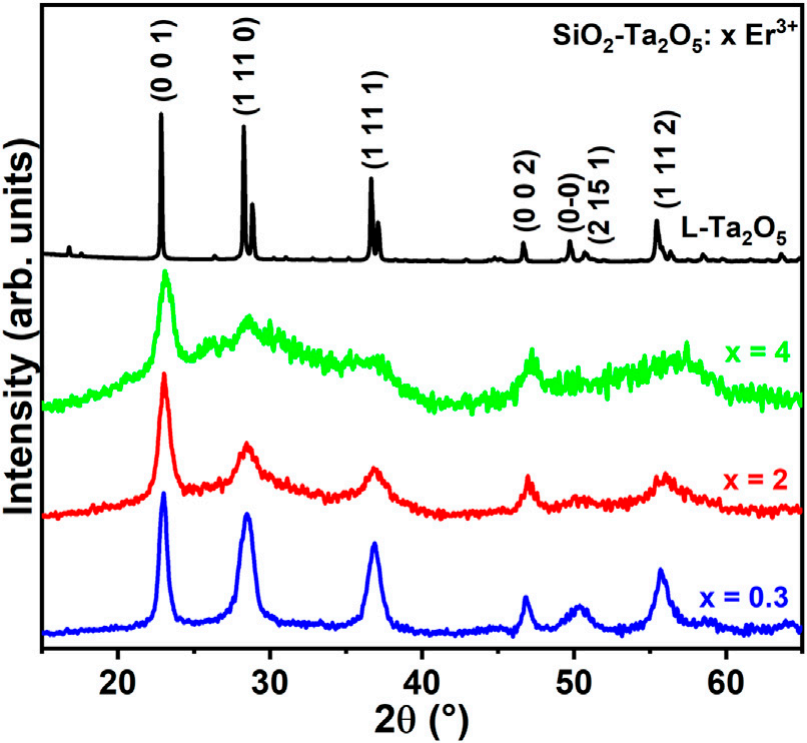
Powder XRD patterns of 0.3, 2.0, and 4.0 mol% Er^3+^-doped SiO_2_-Ta_2_O_5_ nanocomposites calcined at 1100°C for 8 h.

Figure 2 depicts the powder XRD patterns of the Er^3+^-doped SiO_2_-Ta_2_O_5_ nanocomposites doped with 0.3, 2.0, and 4.0 mol% Er^3+^ concentrations. The powder XRD peak positions and relative intensities of all the Er^3+^-doped nanocomposites agreed well with the peak positions and relative intensities reported for the orthorhombic L-Ta_2_O_5_ system. The powder XRD of Er^3+^-doped SiO_2_-Ta_2_O_5_ exhibited amorphous SiO_2_ halos localized between 2θ = 15 and 40°. We did not detect any additional reflection peak, which indicated the absence of secondary phases composed by Er^3+^ ions, at least within the detection limits of this technique. The L-Ta_2_O_5_ powder XRD peaks enlarged with increasing Er^3+^ concentration, pointing to the influence of Er^3+^ insertion on nanocomposite crystallinity.

Bragg peak broadening in diffractograms can be related to structural factors, such as microstrains and crystallite domain sizes. To evaluate the changes caused by the different Er^3+^ doping levels, we estimated the average crystallite sizes of the Ta_2_O_5_ crystals dispersed in the amorphous silica host by using the Scherrer’s formula. This formula is described by t = βλ/Bcosθ, where t is the average crystallite size, β is the Scherrer constant related to the crystallite morphology (is assumed 0.89 for spherical crystallites), λ is the X-Ray wavelength (in Å), B is the integral breadth, and θ is the diffraction angle (in rad) of the peak used in this calculation(Patterson, 1939). Table 2 lists the calculated average crystallite sizes for the 0.3, 2.0, and 4.0 mol% Er^3+^-doped SiO_2_-Ta_2_O_5_ nanocomposites as well as undoped Ta_2_O_5_.

**TABLE 2:** Average crystallite diameters of undoped Ta_2_O_5_ and 0.3, 2.0, and 4.0 mol% Er^3+^-doped SiO_2_-Ta_2_O_5_ nanocomposites calcined at 1100°C for 8 h.

Sample	Average crystallite size (nm)
Undoped Ta_2_O_5_	20.4
SiO_2_-Ta_2_O_5_: 0.3% Er^3+^	9.9
SiO_2_-Ta_2_O_5_: 2.0% Er^3+^	6.7
SiO_2_-Ta_2_O_5_: 4.0% Er^3+^	4.0

**FIGURE 3:**
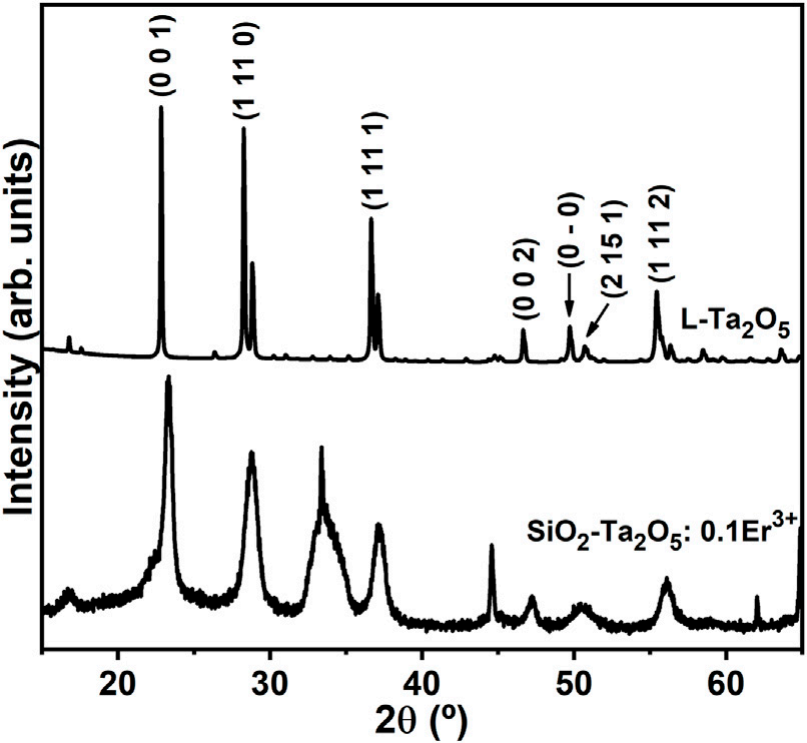
Powder XRD patterns of the 0.1 mol% Er^3+^-doped SiO_2_-Ta_2_O_5_ densified film and pure commercial L-Ta_2_O_5_ powder.

The crystallite size calculations were performed based on the reflection peaks assigned to the (0 0 1), (1 11 0) and (1 11 1) planes of the L-Ta_2_O_5_ crystalline phase. From the diffractogram data it is possible to observe a drastic reduction in the intensity of the powder XRD peaks as well as their broadening. The crystallite dimensions decreased from 9.9 to 4.0 nm as the Er^3+^ doping level increases. RE^3+^ addition to the SiO_2_-Ta_2_O_5_ systems generated additional site distortions and defects, reducing long-distance packing and lowering the degree of crystallinity of the systems. The RE^3+^ dopant promoted a restricting force on the host grain boundaries. If we consider that the restricting forces were greater than the driving forces for grain growth, the grain boundary motion could be hindered, so that crystallite sizes decreased with increasing amount of RE^3+^ dopant, agreeing with our findings for the triply doped SiO_2_-Ta_2_O_5_ nanocomposites. The same crystallite size changing as a function of RE^3+^ insertion has been reported for Sm^3+^-doped ZnO thin films (He et al., 2015; Kayani et al., 2020), Er^3+^-doped Y_2_O_3_ obtained by polymeric precursor (Perrella et al., 2014), and Er^3+^-doped NiO-based nanoparticles (Shkir, 2020), among others. The larger RE^3+^ ionic radii compared to the radii of the metal ions in these hosts produced strains in the matrix lattice, reducing further crystal growth. We attributed the powder XRD pattern of the 0.1 mol% Er^3+^-doped SiO_2_-Ta_2_O_5_ densified planar waveguide (Figure 3) to reflection planes of the orthorhombic L-Ta_2_O_5_ crystalline phase, with the presence of an additional peak at 33°, ascribed to the Si (100) substrate. This diffraction profile indicated that L-Ta_2_O_5_ crystals were dispersed in the amorphous silica host, as reported previously for analogous SiO_2_-Ta_2_O_5_ nanocomposites.

**FIGURE 4:**
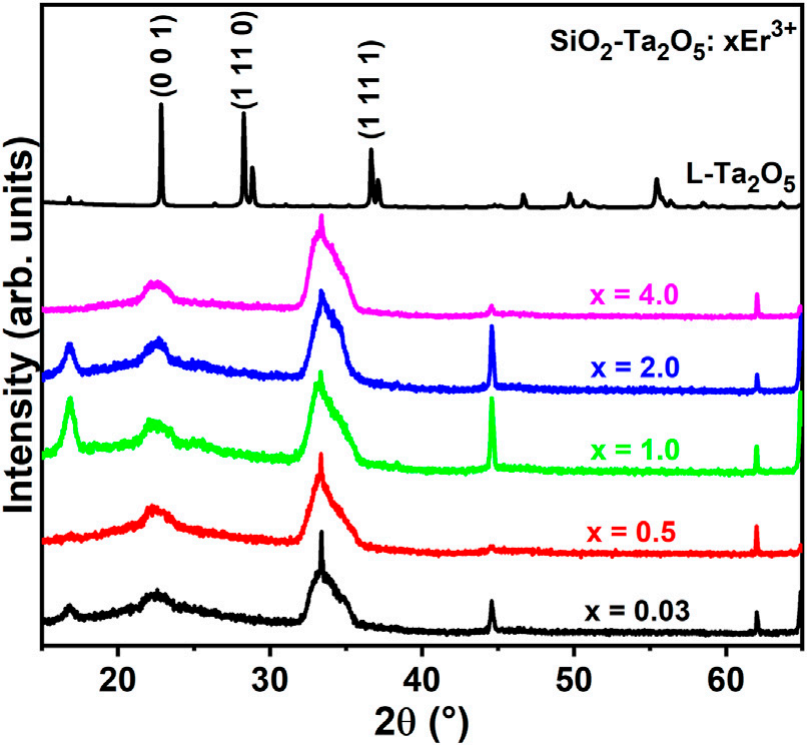
Powder XRD patterns of the 0.03, 0.5, 1.0, 2.0, and 4.0 mol% Er^3+^-doped SiO_2_-Ta_2_O_5_ films.

Figure 4 displays the powder XRD patterns of the planar waveguides prepared with several Er^3+^ doping concentrations. All the powder XRD patterns showed the characteristic L-Ta_2_O_5_ diffraction peak at 23°, attributed to the (0 0 1) plane, attesting the crystallization of Ta_2_O_5_ nanoparticles into the silica-based host. The other powder XRD peaks of the orthorhombic L-Ta_2_O_5_ crystalline phase are not well pronounced due to the intense diffraction signals assigned to the silica on silicon (1 0 0) substrate.

**FIGURE 5:**
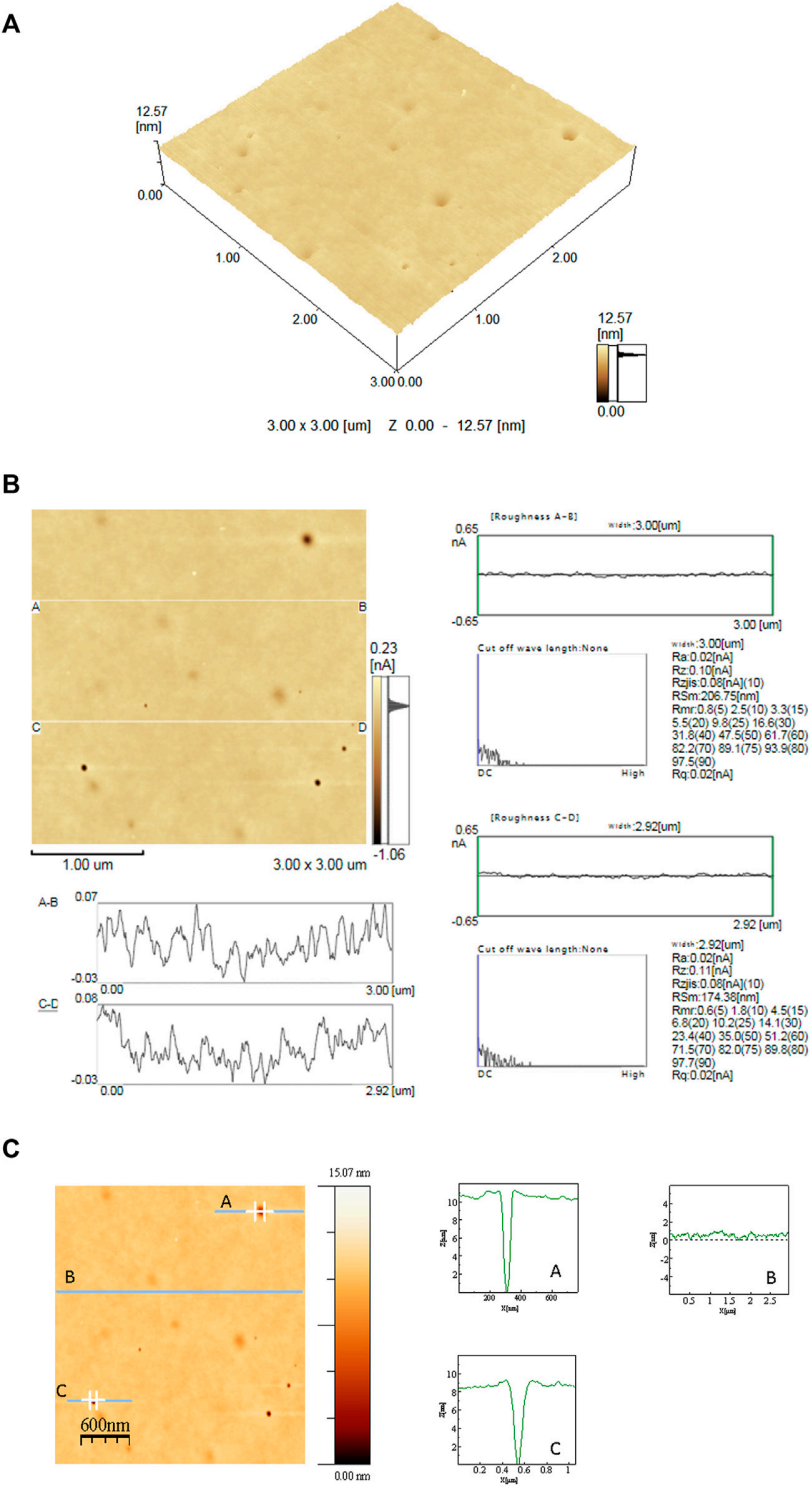
3D **(A)** and 2D **(B)** AFM topographical images, obtained in the contact mode, of the 0.03% Er^3+^-doped SiO_2_-Ta_2_O_5_ multilayered film prepared by the sol-gel route and deposited on a SiO_2_/Si substrate. **(C)** 2D AFM topographical image obtained in the contact mode of drawing profile lines the 0.03% Er^3+^-doped SiO_2_-Ta_2_O_5_ multilayered film prepared by the sol-gel route, deposited on a SiO_2_/Si substrate.

The planar waveguide surfaces were evaluated by atomic force microscopy (AFM) using the contact and tapping scan modes. Figure 5 shows a typical surface image of the 0.03 mol% of Er^3+^-doped SiO_2_-Ta_2_O_5_ film, which is crack-free and has a smooth surface with low roughness mean surface (Ra) of 0.205 nm. The low Ra value confirms a high optical quality of the film, as required for waveguide application, where the roughness of the surface represents a significant source of losses during light propagation. Figure 5B illustrates roughness analyses, which indicate not only a homogeneous surface but also a low content of surface defects in small depths. The presence of defects was identified on film surface by drawing profile lines on the topographical image (Figure 5C).

Figure 5C illustrates the depth profiles of the segments displayed in the 2D AFM image. Considering the surface roughness in the A and C traces, we observed pores with a minimum height of 8 nm and diameters between 39 and 137 nm.

**FIGURE 6:**
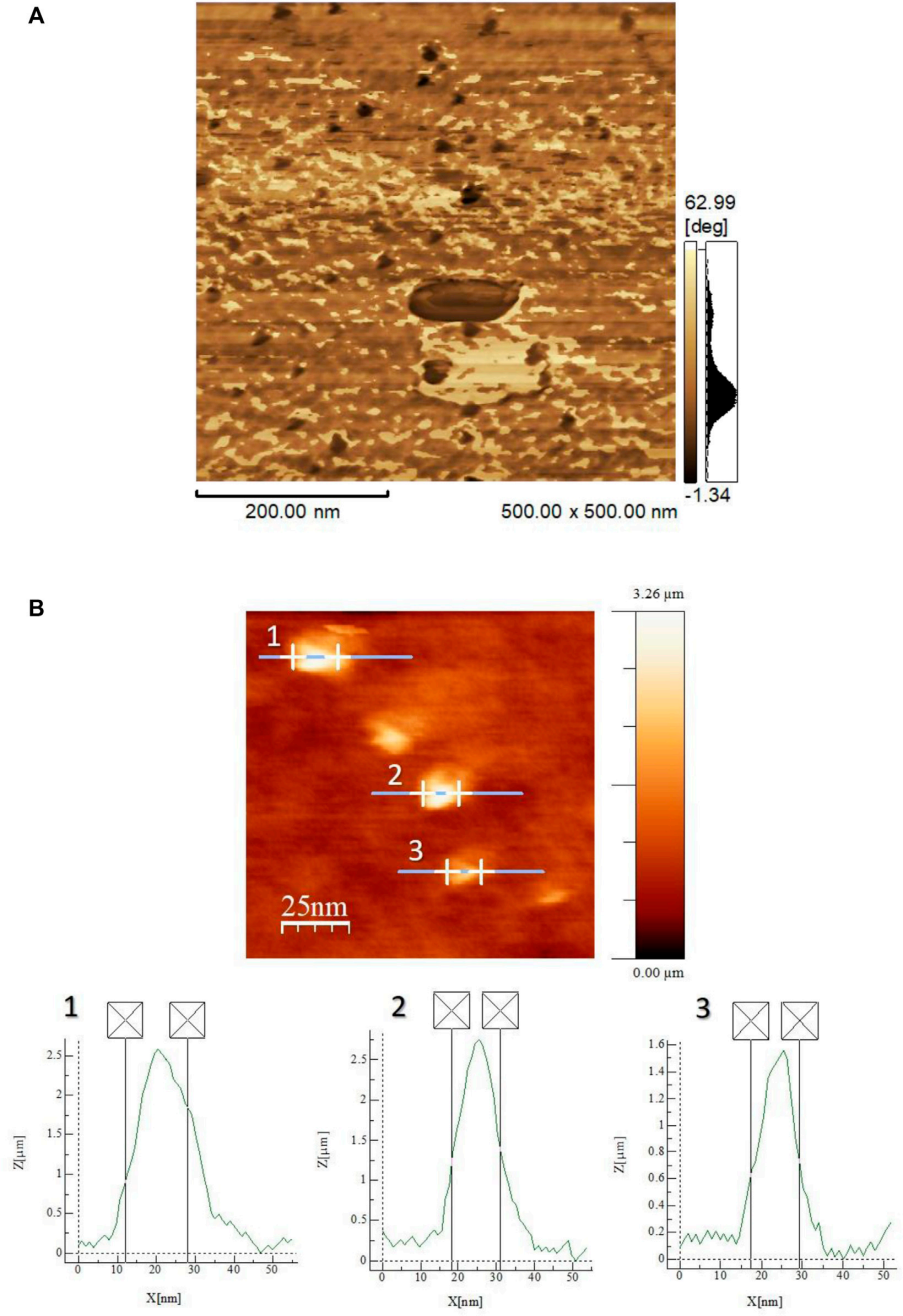
AFM phase-contrast image **(A)** and 2D AFM topographical image obtained in the dynamic mode and the bright spot dimension graphs **(B)** of the 0.03% Er^3+^-doped SiO_2_-Ta_2_O_5_ multilayered film prepared by the sol–gel route, deposited on a SiO_2_/Si substrate.

The purpose of using the dynamic mode is to map differences in viscoelasticity on the sample surface, which allows the Ta_2_O_5_ nanocrystals to be detected on the SiO_2_ amorphous host (Pang et al., 2000). The phase images obtained by scanning the film in the dynamic mode show the regions with low viscoelasticity composition, corresponding to the Ta_2_O_5_ nanocrystals, in darker tones than the silica-based host (Figure 6).

Figure 6B displays the 2D AFM topographical image obtained in the dynamic mode of the 0.03% Er^3+^-doped SiO_2_-Ta_2_O_5_ planar waveguide. We attributed the bright regions in the topographical images (Figure 6B) of this glass ceramic matrix to superficial Ta_2_O_5_ nanocrystals and/or aggregates. The bright spot diameters ranged from 12 to 16 nm. These Ta_2_O_5_ dimensions agreed with the crystallite size values calculated from the most intense L-Ta_2_O_5_ diffraction peak of the Er^3+^-doped SiO_2_-Ta_2_O_5_ nanocomposites. It is important to emphasize that usually the characterization of films using techniques such as transmission electron microscopy to observe the formation of nanocrystals requires complex techniques for sample preparation with high cost and long time. In this work, an AFM was applied mapping the sample according to viscoelastic properties. This allowed the observation of the formation of tantalum oxide nanocrystals distributed in the homogeneous silica-based matrix.

### Photoluminescent Properties

**FIGURE 7:**
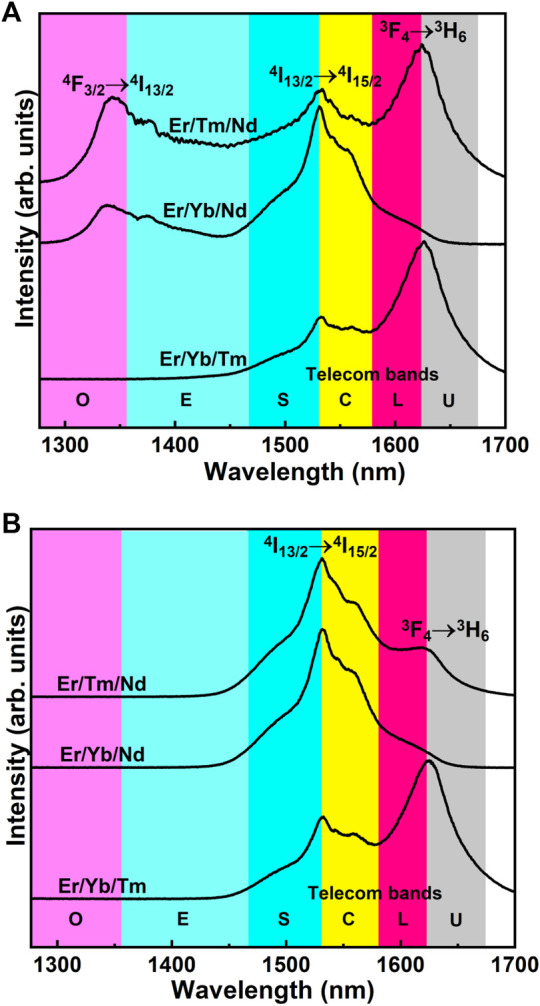
PL emission spectra of triply doped SiO_2_-Ta_2_O_5_ nanocomposites under excitation at **(A)** 808 nm and **(B)** 980 nm.

**FIGURE 8:**
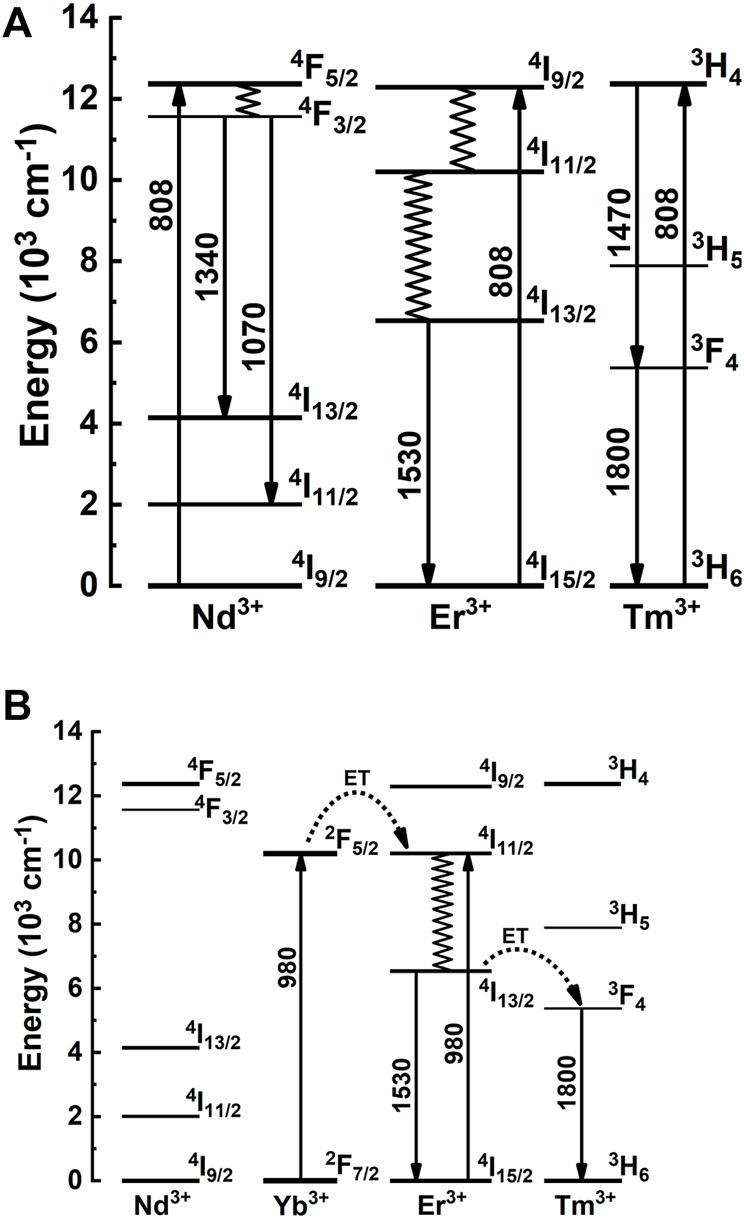
Partial energy levels diagram of the Nd^3+^, Er^3+^ and Tm^3+^ ions under excitation at 808 nm **(A)**, and Yb^3+^, Er^3+^, Tm^3+^ ions under excitation at 980 nm **(B)** and possible excitation/emission mechanisms.

Figure 7 shows the PL emission spectra of the triply doped SiO_2_-Ta_2_O_5_ nanocomposites in the NIR region under excitation at 808 and 980 nm. Intense broad NIR emission band was detected, with maximum positioned around 1.3-, 1.5-, and 1.65-µm regions, assigned to the ^4^F_3/2_→^4^I_13/2_, ^4^I_13/2_→^4^I_15/2_, and ^3^F_4_→^3^H_6_ transitions of the Nd^3+^, Er^3+^, and Tm^3+^ ions, respectively. Figure 8 displays the diagram of partial energy levels of the Er^3+^, Yb^3+^, Tm^3+^, and Nd^3+^ ions and the possible excitation/emission mechanisms involved in the intraconfigurational f-f transitions in the NIR region. Table 3 lists the full-width at half-maximum (FWHM) values of the bands centered in the 1.5-µm region. The most satisfactory results concerning broad NIR emission emerge upon excitation at 808 nm, with FWHM value of 173 (±5 nm), which has been observed for the Er^3+^/Tm^3+^/Nd^3+^ doped SiO_2_-Ta_2_O_5_ nanocomposite. The FWHM values obtained in the present work are lower than ones obtained for some RE-doped tellurite glass (Rivera et al., 2014; Hou et al., 2021). However, they are similar to others presented in the literature (Han et al., 2014; Shen et al., 2020a). The advantage of using SiO_2_-Ta_2_O_5_ based materials is related to their low toxicity, easy preparation, stability under different chemical and physical conditions, not mention the optical properties of silica.

**TABLE 3:** FWHM values of triply doped SiO_2_-Ta_2_O_5_ nanocomposites under excitation at 808 and 980 nm.

Dopant	Central position (nm)		FWHM (±5 nm)	
	λ_exc_ = 808 nm	λ_exc_ = 980 nm	λ_exc_ = 808 nm	λ_exc_ = 980 nm
Er^3+^/Tm^3+^/Nd^3+^	1533.5	1531.0	173	65
Er^3+^/Yb^3+^/Nd^3+^	1531.0	1531.5	54	57
Er^3+^/Yb^3+^/Tm^3+^	1532.5	1532.5	156	148

The inhomogeneous broadening of all the NIR emission spectra demonstrated that the Er^3+^, Nd^3+^, and Tm^3+^ ions were preferentially located in different symmetry sites of the orthorhombic L-Ta_2_O_5_ structure, as observed previously by Er^3+^ doped SiO_2_-Ta_2_O_5_ nanocomposite (; Ferrari et al., 2011a and Ferrari et al., 2011b).

**FIGURE 9:**
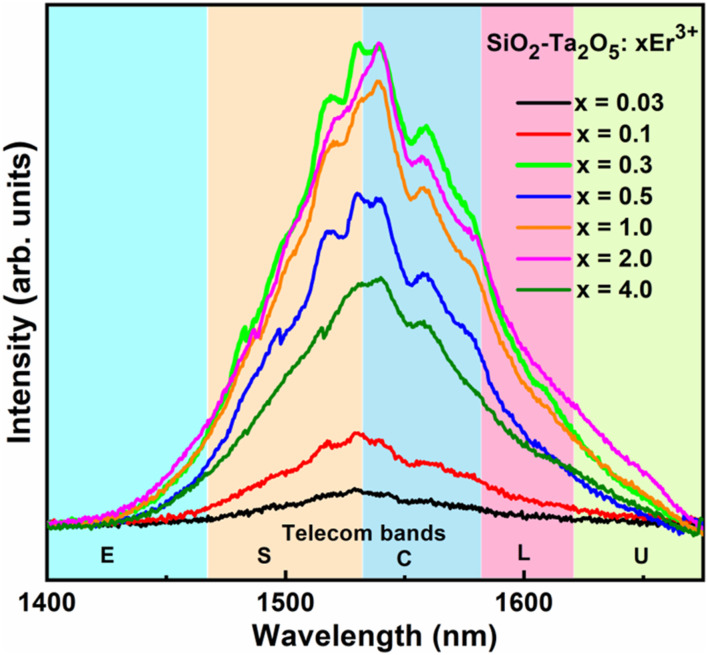
Emission spectra in the infrared region of the 0.03, 0.1, 0.3, 0.5, 1, 2, and 4 mol% Er^3+^-doped SiO_2_-Ta_2_O_5_ nanocomposites calcined at 1100°C for 8 h, under excitation at 980 nm with 100-mW power pump.

Figure 9 shows the emission spectra in the infrared range of the SiO_2_-Ta_2_O_5_ nanocomposites doped with several Er^3+^ concentrations. The broadbands are assigned to the Er^3+^:^4^I_13/2_→^4^I_15/2_ transition, with maximum emission centered around 1534 nm and bandwidths between 77 and 93 nm (Table 4). These bandwidth values were larger compared to other Er^3+^-doped matrixes reported in the literature:Er^3+^-doped silica-hafnia, with bandwidth of 48 nm (Gonçalves et al., 2002); Er^3+^:SiO_2_-Al_2_O_3_, with bandwidth of 47 nm (Benatsou et al., 1997), Er^3+^-doped niobic tellurite glass (Lin et al., 2003); Er^3+^, Yb^3+^:Bi_2_ZnB_2_O_7_ glass-ceramic, with bandwidth of 84 nm (Li et al., 2019); and Er^3+^:SiO_2_-Ta_2_O_5_ nanocomposite films, with bandwidth of 64 nm (Ferrari et al., 2010), among others.

**TABLE 4:** Values of central positions and bandwidths of the ^4^I_13/2_ → ^4^I_15/2_ Er^3+^ transition for Er^3+^-doped SiO_2_-Ta_2_O_5_ nanocomposites doped with different Er^3+^ concentrations, annealed at 1100°C for 8 h.

Mol% Er^3+^	Central position (nm)	FWHM (±5 nm)
0.03	1528.5	77
0.1	1530.5	85
0.3	1531.0	93
0.5	1530.0	92
1.0	1538.5	90
2.0	1539.0	91
4.0	1540.0	90

The nanocomposite 0.3 mol% Er^3+^-doped SiO_2_-Ta_2_O_5_ have the broadest NIR emission, with FWHM of 93 (±3 nm). Table 4 depicts the central position and inhomogeneous broadening values of the nanocomposites doped with different Er^3+^ concentrations. Inhomogeneous broadening of the 0.03 and 0.1 mol% Er^3+^-doped SiO_2_-Ta_2_O_5_ nanocomposites was relatively narrower compared to the other nanocomposites. It was clearly observed that for higher concentrations there was an FWHM values increase to 90 nm (±3 nm).

The 4.0 mol% Er^3+^-doped SiO_2_-Ta_2_O_5_ nanocomposite had an intermediate relative intensity due to luminescence quenching, which can be associated with non-radiative processes, such as energy migration and up-conversion. This observation will be confirmed by the lifetime values.

The high RE^3+^ doping level can promote cluster formation, which contributes to energy losses via non-radiative processes (Auzel and Goldner, 2001). Therefore, this type of energy transfer mechanism via a non-radiative process can be controlled according to the ion concentration and its distribution into the host.

**FIGURE 10:**
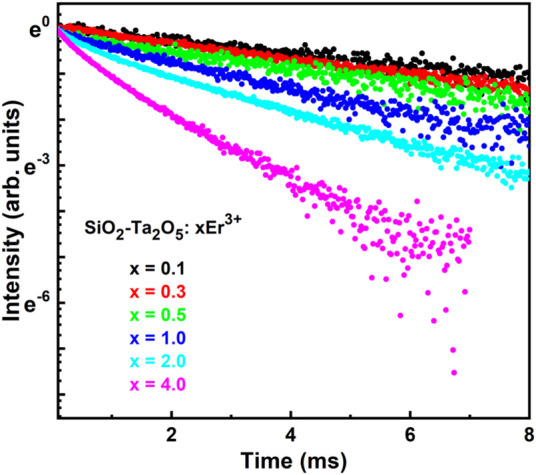
Photoluminescence decay curves of the ^4^I_13/2_ → ^4^I_15/2_ transition of Er^3+^ ions in the 0.1, 0.3, 0.5, 1.0, 2.0, and 4.0 mol% Er^3+^-doped SiO_2_-Ta_2_O_5_-based waveguides, obtained by using 100 mW excitation power at 980 nm.

As previously reported for Er^3+^-doped SiO_2_-Ta_2_O_5_ systems, RE^3+^ ions are preferentially distributed in a rich Ta_2_O_5_ environment. Such inhomogeneous broadenings can be attributed to the particular orthorhombic L-Ta_2_O_5_ crystalline phase, which presents many different symmetry sites, more precisely 12 sites where Er^3+^ ions can substitute the Ta^5+^ ions and/or occupy interstitial spaces of the Ta_2_O_5_ structure (Ferrari et al., 2011b). To study the presence of clusters and non-radiative luminescence-suppressing processes, luminescence decay curves were collected and the lifetime of the ^4^I_13/2_ excited state of the Er^3+^ ions was calculated as a function of the concentration of the dopant ions. NIR photoluminescence decay curves are displayed in Figure 10.

Figure 10 shows that the NIR photoluminescence decay curves of the planar waveguides with Er^3+^ doping level up to 0.5 mol% could be well fitted by a single exponential function. Above 0.5 mol%, luminescence quenching occurred due to non-radiative process competition. This deviation from the mono-exponential decay could be related to the presence of RE clusters, which indicates to be increased for higher Er^3+^ concentration. The value of 1/e to estimate the ^4^I_13/2_ excited state measured lifetime values were here used as displayed in Table 5.

**TABLE 5:** ^4^I_13/2_ emitting level lifetimes of Er^3+^-doped SiO_2_-Ta_2_O_5_ planar waveguides for different Er^3+^ concentrations.

Mol% Er^3+^	τ_1/e_ (ms)
0.1	6.90
0.3	5.90
0.5	5.20
1.0	2.80
2.0	1.80
4.0	0.93

**FIGURE 11:**
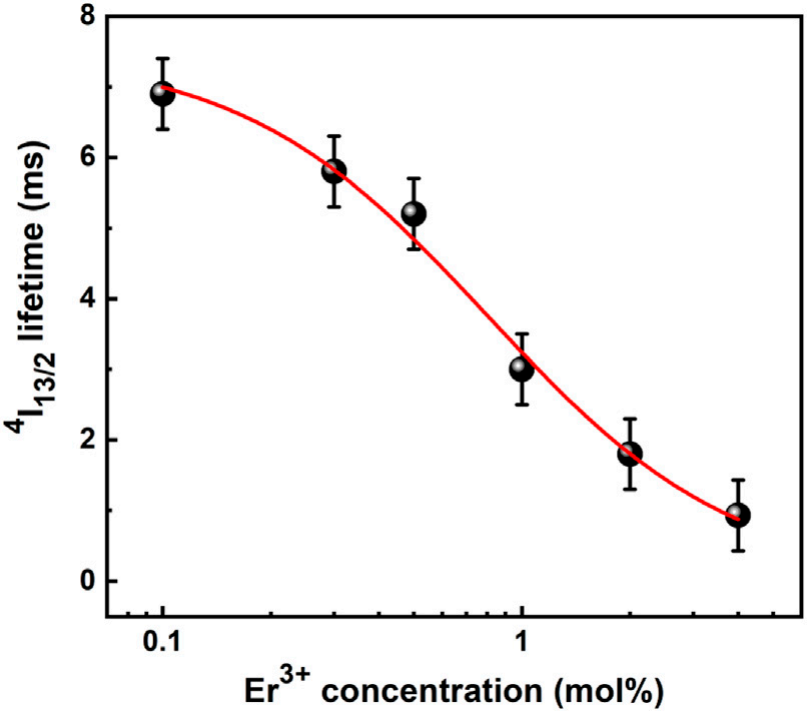
Lifetime values of the ^4^I_13/2_ → ^4^I_15/2_ transition of Er^3+^-doped SiO_2_-Ta_2_O_5_ based waveguides as a function of Er^3+^ doping concentrations (from 0.1 to 4.0 mol%). The best fit is represented by the red line.

The lifetime values decreased as Er^3+^ doping level increases, as it can be seen in Figure 11. The high Er^3+^ concentration shortened the distance between the dopant ions distributed in the matrix, forming a cluster and promoting a non-radiative process involving energy migration. The presence of defects in the host structure caused the phonons and the dopant ions to interact, promoting electron-hole pair recombination with deactivation of the Er^3+^ excited state. Similar curve of ^4^I_13/2_ Er^3+^ lifetime values versus dopant concentration has been reported for Er^3+^-doped SiO_2_-HfO_2_ planar waveguides as a function of Er^3+^ concentration from 0.01 to 4.0 mol%, in which the lifetime decreased from 6.7 to 1.1 ms (Gonçalves et al., 2004). These ^4^I_13/2_ emitting level lifetime behaviors as a function of the Er^3+^ concentration, the so-called quenching concentration, are described by the following empirical equation (Orignac et al., 1999; Gonçalves et al., 2004):

$$\tau_{obs}=\frac{\tau_{0}}{1+{\left(r/Q\right)}^{p}}\;$$ (1)

where *τ*_*obs*_ is the observed emitting level lifetime, *τ*_*0*_ is the ideal emitting level lifetime in the limit of zero concentration, *r* is the Er^3+^ concentration, *Q* is the quenching concentration, and *p* is a phenomenological parameter characterizing the steepness of the corresponding quenching curve.

Figure 11 shows the measured luminescence lifetime of the ^4^I_13/2_ Er^3+^ metastable state for the Er^3+^-doped SiO_2_-Ta_2_O_5_ films as a function of Er^3+^ concentration. Fitting the experimental data to the empirical Eq. 1, we obtained the following parameters for the best fit represented by the red line: τ_0_= 7.49 ms, Q= 0.81 mol%, and *p* = 1.26. The Er^3+^-doped SiO_2_-Ta_2_O_5_ films exhibited higher quenching concentration compared to other materials. In particular, the quenching concentration of 0.81 mol% was relatively higher than the value of 0.62 mol% reported for the Er^3+^/Yb^3+^-codoped 80SiO_2_–20TiO_2_–zAl_2_O_3_ (Orignac et al., 1999) and equal to the value of 0.81 mol% described for Er^3+^-doped 70SiO_2_-30HfO_2_ (Gonçalves et al., 2004), proving that tantalum oxide is a promising host for RE ions for NIR broadband emission.

## Conclusion

In this work, the potential for obtaining broad and super broadband NIR emission from silicate systems containing tantalum oxide nanocrystals doped with rare earth ions was proved, showing a set of optimal features making them promising candidates for active planar waveguides operating in wide range of telecom band.

RE^3+^- doped SiO_2_-Ta_2_O_5_ nanocomposites and planar waveguides were prepared by the sol–gel method and the dip-coating technique, for film deposition. XRD analysis revealed the presence of L-Ta_2_O_5_ nanocrystals dispersed in the SiO_2_ amorphous host. High RE^3+^ concentrations can reduce the crystallinity and the crystallite sizes of the Ta_2_O_5_ in SiO_2_-Ta_2_O_5_ nanocomposites and waveguides. AFM revealed uniform surface with roughness in the order of 0.2 nm, attesting to the excellent quality of these waveguides for optical applications give those optical losses by light scattering are avoided. All the SiO_2_-Ta_2_O_5_ nanocomposites display broadband emission, with maximum at 1534 nm and bandwidths ranging from 77 to 93 nm for Er^3+^-doped and up to 173 nm for Er^3+^/Tm^3+^/Nd^3+^-doped. The Er^3+ 4^I_13/2_ lifetimes of the Er^3+^-doped SiO_2_-Ta_2_O_5_ waveguides diminish as a function of the Er^3+^ content. Emission quenching of the Er^3+^-doped SiO_2_-Ta_2_O_5_ based waveguides due to the Er^3+^ doping level is negligible up to 0.81 mol%. Such properties make the present materials interesting systems for applications in photonics.

## Data Availability

The original contributions presented in the study are included in the article, further inquiries can be directed to the corresponding author.

## References

[B1] Aquino F. T. Caixeta F. J. de Oliveira Lima K. Kochanowicz M. Dorosz D. Gonçalves R. R. (2018). Broadband NIR Emission from Rare Earth Doped-SiO2-Nb2O5 and SiO2-Ta2O5 Nanocomposites. J. Luminescence 199, 138–142. 10.1016/j.jlumin.2018.03.018

[B2] Auzel F. Goldner P. (2001). Towards Rare-Earth Clustering Control in Doped Glasses. Opt. Mater. 16, 93–103. 10.1016/S0925-3467(00)00064-1

[B3] Benatsou M. Capoen B. Bouazaoui M. Tchana W. Vilcot J. P. (1997). Preparation and Characterization of Sol-Gel Derived Er3+: Al2O3-SiO2 Planar Waveguides. Appl. Phys. Lett. 71, 428–430. 10.1063/1.119569

[B4] Cisco (2020). Annual Internet Report (2018–2023). Available online: https://www.cisco.com/c/en/us/solutions/collateral/ executive-perspectives/annual-internet-report/white-paper-c11-741490.html (Accessed on February 14, 2022).

[B5] Dan H. K. Le D.-N. Nguyen-Truong H. T. Tap T. D. Vinh H. X. Ty N. M. (2020). Effects of Y3+on the Enhancement NIR Emission of Bi+-Er3+ Co-doped in Transparent Silicate Glass-Ceramics for Erbium-Doped Fiber Amplifier (EDFA). J. Luminescence 219, 116942. 10.1016/j.jlumin.2019.116942

[B6] Ferrari J. L. Lima K. D. O. Gonçalves R. R. (2021). Refractive Indexes and Spectroscopic Properties to Design Er3+-Doped SiO2-Ta2O5 Films as Multifunctional Planar Waveguide Platforms for Optical Sensors and Amplifiers. ACS OMEGA 6, 8784–8796. 10.1021/acsomega.0c05351 33842750 PMC8028007

[B39] Desurvire E. Simpson J. R. Becker P. C. (1987). High-Gain Erbium-Doped Traveling-Wave Fiber Amplifier. Opt. Lett. 12, 888. 10.1364/ol.12.000888 19741905

[B7] Ferrari J. L. Lima K. O. Maia L. J. Q. Gonçalves R. R. (2010). Sol-gel Preparation of Near-Infrared Broadband Emitting Er3+-Doped SiO2-Ta2O5 Nanocomposite Films. Thin Solid Films 519, 1319–1324. 10.1016/j.tsf.2010.09.035

[B8] Ferrari J. L. Lima K. O. Maia L. J. Q. Ribeiro S. J. L. Gomes A. S. L. Gonçalves R. R. (2011a). Broadband NIR Emission in Sol-Gel Er3+-Activated SiO2-Ta2O5 Glass Ceramic Planar and Channel Waveguides for Optical Application. J. Nanosci. Nanotech. 11, 2540–2544. 10.1166/jnn.2011.3565 21449421

[B9] Ferrari J. L. Lima K. O. Maia L. J. Q. Ribeiro S. J. L. Gonçalves R. R. (2011b). Structural and Spectroscopic Properties of Luminescent Er3+-Doped SiO2-Ta2O5 Nanocomposites. J. Am. Ceram. Soc. 94, 1230–1237. 10.1111/j.1551-2916.2010.04191.x

[B10] Gonçalves R. R. Carturan G. Montagna M. Ferrari M. Zampedri L. Pelli S. (2004). Erbium-activated HfO2-Based Waveguides for Photonics. Opt. Mat. (Amst). 25, 131–139. 10.1016/S0925-3467(03)00261-1

[B11] Gonçalves R. R. Carturan G. Zampedri L. Ferrari M. Montagna M. Chiasera A. (2002). Sol-gel Er-Doped SiO2-HfO2 Planar Waveguides: A Viable System for 1.5 μm Application. Appl. Phys. Lett. 81, 28–30. 10.1063/1.1489477

[B12] Han X. Shen L. Pun E. Y. B. Ma T. Lin H. (2014). Pr3+-doped Phosphate Glasses for Fiber Amplifiers Operating at 1.38-1.53μm of the Fifth Optical Telecommunication Window. Opt. Mater. 36, 1203–1208. 10.1016/j.optmat.2014.02.032

[B13] He H. Y. Fei J. Lu J. (2015). Sm-doping Effect on Optical and Electrical Properties of ZnO Films. J. Nanostruct Chem. 5, 169–175. 10.1007/s40097-015-0147-0

[B14] Herrera A. Londoño F. Balzaretti N. M. (2021). Structural and Optical Properties of Nd3+ Doped GeO2-PbO Glass Modified by TiO2 for Applications in Laser and Fiber Amplifier. Opt. Mater. 113, 110884–110889. 10.1016/j.optmat.2021.110884

[B15] Hou G. Cao L. Zhang C. Yu X. Fu W. Li G. (2021). Improvement of Ultra-broadband Near-Infrared Emission in Nd3+-Er3+-Pr3+ Tri-doped Tellurite Glasses. Opt. Mater. 111, 110547. 10.1016/j.optmat.2020.110547

[B16] Huang L. Shen S. Jha A. (2004). Near Infrared Spectroscopic Investigation of Tm3+-Yb3+ Co-doped Tellurite Glasses. J. Non-Crystalline Solids 345-346, 349–353. 10.1016/j.jnoncrysol.2004.08.042

[B17] Kayani Z. N. Sahar M. Riaz S. Naseem S. Saddiqe Z. (2020). Enhanced Magnetic, Antibacterial and Optical Properties of Sm Doped ZnO Thin Films: Role of Sm Doping. Opt. Mater. 108, 110457. 10.1016/j.optmat.2020.110457

[B18] Khamis M. A. Ennser K. (2018). “Comparative Studies of Thulium and Erbium-Doped Fiber Amplifiers for Dynamic Optical WDM Networks,” in 2018 IEEE British and Irish Conference on Optics and Photonics (BICOP), London, UK, 12-14 December 2018, 1–4. 10.1109/BICOP.2018.8658309

[B19] Li G. S. Zhang C. M. Zhu P. F. Jiang C. Song P. Zhu K. (2016). Broadband Near-Infrared Emission in Pr3+-Er3+ Codoped Phosphate Glasses for Optical Amplifiers. Ceram. Int. 42, 5558–5561. 10.1016/j.ceramint.2015.12.026

[B20] Li M. Luan J. Zhang Y. Jiang F. Zhou X. Tang J. (2019). Spectroscopic Properties of Er/Yb Co-doped Glass Ceramics Containing Nanocrystalline Bi2ZnB2O7 for Broadband Near-Infrared Emission. Ceram. Int. 45, 18831–18837. 10.1016/j.ceramint.2019.06.116

[B21] Lin H. Meredith G. Jiang S. Peng X. Luo T. Peyghambarian N. (2003). Optical Transitions and Visible Upconversion in Er3+ Doped Niobic Tellurite Glass. J. Appl. Phys. 93, 186–191. 10.1063/1.1527209

[B22] Martins M. M. Kassab L. R. P. da Silva D. M. de Araújo C. B. (2019). Tm3+ Doped Bi2O3-GeO2 Glasses with Silver Nanoparticles for Optical Amplifiers in the Short-Wave-Infrared-Region. J. Alloys Compd. 772, 58–63. 10.1016/j.jallcom.2018.08.146

[B40] Mears R. J. Reekie L. Jauncey I. M. Payne D. N. (1987). Low-Noise Erbium-Doped Fibre Amplifier Operating at 1.54 μm. Electron. Lett. 23, 1026. 10.1049/el:19870719

[B23] Meng X.-g. Qiu J.-r. Peng M.-y. Chen D.-p. Zhao Q.-z. Jiang X.-w. (2005). Near Infrared Broadband Emission of Bismuth-Doped Aluminophosphate Glass. Opt. Express 13, 1628–1634. 10.1364/OPEX.13.001628 19495038

[B24] Muscelli W. C. Lima K. d. O. Thomaz Aquino F. Gonçalves R. R. (2016). Blue and NIR Emission from Nanostructured Tm3+/ Yb3+co-Doped SiO2-Ta2O5for Photonic Applications. J. Phys. D. Appl. Phys. 49, 175107–175111. 10.1088/0022-3727/49/17/175107

[B25] Orignac X. Barbier D. Min X. Almeida R. M. Mccarthy O. Yeatman E. (1999). Sol–gel Silica/titania-On-Silicon Er/Yb-Doped Waveguides for Optical Amplification at 1.5 μm. Opt. Mater. 12, 1–18. 10.1016/S0925-3467(98)00076-7

[B26] Pang G. K. H. Baba-Kishi K. Z. Patel A. (2000). Topographic and Phase-Contrast Imaging in Atomic Force Microscopy. Ultramicroscopy 81, 35–40. 10.1016/S0304-3991(99)00164-3 10998788

[B27] Patterson A. L. (1939). The Scherrer Formula for X-Ray Particle Size Determination. Phys. Rev. 56, 978–982. 10.1103/PhysRev.56.978

[B28] PCH (2021). Packet Clearing House, Internet Exchange Point Directory Reports. Available online: http://www.pch.net/ixp/summary (accessed on February 14, 2022).

[B29] Perrella R. V. Dos Santos D. P. Poirier G. Y. Góes M. S. Ribeiro S. J. L. Schiavon M. A. (2014). Er3+-doped Y2O3 Obtained by Polymeric Precursor: Synthesis, Structure and Upconversion Emission Properties. J. Luminescence 149, 333–340. 10.1016/j.jlumin.2014.01.052

[B30] Rivera V. A. G. El-Amraoui M. Ledemi Y. Messaddeq Y. Marega E. (2014). Expanding Broadband Emission in the Near-IR via Energy Transfer between Er3+-Tm3+ Co-doped Tellurite-Glasses. J. Luminescence 145, 787–792. 10.1016/j.jlumin.2013.08.071

[B31] Shen X. Zhang Y. Xia L. Li J. Yang G. Zhou Y. (2020a). Broadband Flat Near-Infrared Emission from Tellurite Glass Doped with Tm3+, Er3+ and Ag NPs. Opt. Laser Technol. 129, 106264. 10.1016/j.optlastec.2020.106264

[B32] Shen X. Zhang Y. Xia L. Li J. Yang G. Zhou Y. (2020b). Dual Super-broadband NIR Emissions in Pr3+-Er3+-Nd3+ Tri-doped Tellurite Glass. Ceram. Int. 46, 14284–14286. 10.1016/j.ceramint.2020.02.196

[B33] Shkir M. (2020). Noticeable Impact of Er Doping on Structural, Vibrational, Optical, Dielectric and Electrical Parameters of Flash Combustion Synthesized NiO NPs for Optoelectronic Applications. Inorg. Chem. Commun. 121, 108229. 10.1016/j.inoche.2020.108229

[B41] Stephenson N. C. Roth R. S. (1971). Structural Systematics in the Binary System Ta2O5–WO3. V. The Structure of the Low-Temperature Form of Tantalum Oxide L-Ta2O5. Acta Crystallogr. Sect. B Struct. Crystallogr. Cryst. Chem. 27, 1037–1044. 10.1107/s056774087100342x

[B34] Sun H.-T. Zhou J. Qiu J. (2014). Recent Advances in Bismuth Activated Photonic Materials. Prog. Mater. Sci. 64, 1–72. 10.1016/j.pmatsci.2014.02.002

[B35] Terui H. Kobayashi M. (1978). Refractive‐index‐adjustable SiO2‐Ta2O5films for Integrated Optical Circuits. Appl. Phys. Lett. 32, 666–668. 10.1063/1.89848

[B36] Thipparapu N. K. Wang Y. Wang S. Umnikov A. A. Barua P. Sahu J. K. (2019). Bi-doped Fiber Amplifiers and Lasers [Invited]. Opt. Mat. Express 9, 2446. 10.1364/ome.9.002446

[B37] Xia L. Zhang Y. Ding J. Li C. Shen X. Zhou Y. (2021). Er3+/Tm3+/Nd3+ Tri-doping Tellurite Glass with Ultra-wide NIR Emission. J. Alloys Compd. 863, 158626. 10.1016/j.jallcom.2021.158626

[B38] Yang X. L. Wang W. C. Zhang Q. Y. (2018). BaF2 Modified Cr3+/Ho3+ Co-doped Germanate Glass for Efficient 2.0 μm Fiber Lasers. J. Non-Crystalline Solids 482, 147–153. 10.1016/j.jnoncrysol.2017.12.031

